# Recent Advances in the Antihyperlipidemic Activity of Marine Polysaccharides

**DOI:** 10.3390/md24090315

**Published:** 2026-09-09

**Authors:** Yongxuan Liu, Haowei Liu, Dan Li, Shengzhou Ma, Chunxia Li

**Affiliations:** 1 Key Laboratory of Marine Drugs of Ministry of Education, Shandong Key Laboratory of Glycoscience and Glycotherapeutics, School of Medicine and Pharmacy, Ocean University of China, Qingdao 266003, China; 2 Laboratory for Marine Drugs and Bioproducts, Qingdao Marine Science and Technology Center, Qingdao 266237, China; 3 Laboratory of Marine Glycodrugs Research and Development, Marine Biomedical Research Institute of Qingdao, Qingdao 266071, China

**Keywords:** hyperlipidemia, marine polysaccharides, mechanisms of action, structure–activity relationship

## Abstract

Hyperlipidemia is a chronic metabolic disorder in humans, contributing to the onset of cardiovascular diseases (CVDs) that remain the leading cause of death worldwide. Current clinical antihyperlipidemic agents are often accompanied by diverse adverse side effects. Therefore, developing safer novel lipid regulators is an urgent and challenging task. Marine polysaccharides exhibit remarkable antihyperlipidemic activity owing to their distinctive physicochemical properties, multi-target mechanisms, and system-level modulation. This review systematically summarizes recent progress regarding the antihyperlipidemic efficacy of marine polysaccharides and provides comprehensive insights into their sources, structural features, structure–activity relationships, mechanisms of action, and application potential. Furthermore, this review also highlights the existing limitations and challenges in translational research of marine polysaccharides, and puts forward future research directions to advance marine polysaccharides into safe, effective, and sustainable natural therapeutics for hyperlipidemia.

## 1. Introduction

Hyperlipidemia, a chronic metabolic disorder in humans, is characterized by markedly increased levels of circulating lipids and lipoproteins, including low-density lipoproteins (LDLs), very low-density lipoproteins (VLDLs), total cholesterol (TC), triglycerides (TGs), fatty acids and phospholipids, as well as reduced high-density lipoproteins (HDLs) [1,2]. As a major risk factor and predictive indicator, hyperlipidemia contributes to the onset of cardiovascular diseases (CVDs) such as atherosclerosis, coronary heart disease, myocardial infarction, and stroke [3]. CVDs remain the leading cause of death worldwide, accounting for approximately 19.8 million deaths in 2022, or nearly 32% of all deaths globally (https://www.who.int/news-room/fact-sheets/detail/cardiovascular-diseases-(cvds) (accessed on 19 August 2026)). Dyslipidemia is also highly prevalent worldwide, with recent global evidence estimating the prevalence of hypertriglyceridemia and hypercholesterolemia at 28.8% and 24.1%, respectively, while elevated low-density lipoprotein cholesterol (LDL-C) affects approximately 18.9% of adults [4].

Hyperlipidemia can be triggered by multiple factors such as genetics, diet, insulin resistance, inflammation, and hormonal changes, and its underlying mechanisms are highly complicated [5,6]. For example, patients with familial hypercholesterolemia harbor gain-of-function mutations in proprotein convertase subtilisin/kexin type 9 (PCSK9), which are associated with elevated circulating lipid levels [7]. PCSK9 upregulates circulating LDL levels by binding to low-density lipoprotein receptors (LDLRs) on the surface of hepatocytes and promoting LDLR degradation within hepatic lysosomes [8,9]. Numerous hepatic enzymes participate in the synthesis and metabolism of cholesterol and lipids [10,11]. Typical examples include acetyl-CoA carboxylase (ACC), which catalyzes the conversion of acetyl-CoA to malonyl-CoA; lipoprotein lipase, which hydrolyzes TG in chylomicrons and large VLDL; and fatty acid synthase, which mediates the synthesis of long-chain saturated fatty acids. Dysregulated expression of these hepatic enzymes results in lipid metabolic disorders, such as dyslipidemia.

Clinical antihyperlipidemic drugs are divided into TC-lowering, TG-lowering, and other types. Traditional TC-lowering drugs include 3-hydroxy-3-methylglutaryl-coenzyme A reductase (HMG-CoA-R) inhibitors (statins) [12], cholesterol absorption inhibitors (i.e., ezetimibe) [13], and bile acid sequestrants (i.e., cholestyramine) [14]. Traditional TG-lowering drugs consist of fibrates (i.e., bezafibrate) [15], nicotinic acid derivatives (i.e., niacin) [16], and high-purity fish oil formulations (i.e., ethyl eicosapentaenoate) [17]. The remaining categories involve novel lipid-lowering agents, including PCSK9 inhibitors [18], apolipoprotein B100 (apoB100) synthesis inhibitors [19], and microsomal triglyceride transfer protein inhibitors [20]. Many traditional antihyperlipidemic drugs cause various side effects or adverse reactions, such as liver injury, rhabdomyolysis, new-onset diabetes mellitus, nausea, and elevated bleeding risk [21]. Developing safer novel lipid regulators is therefore an urgent and challenging task.

**Figure 1:**
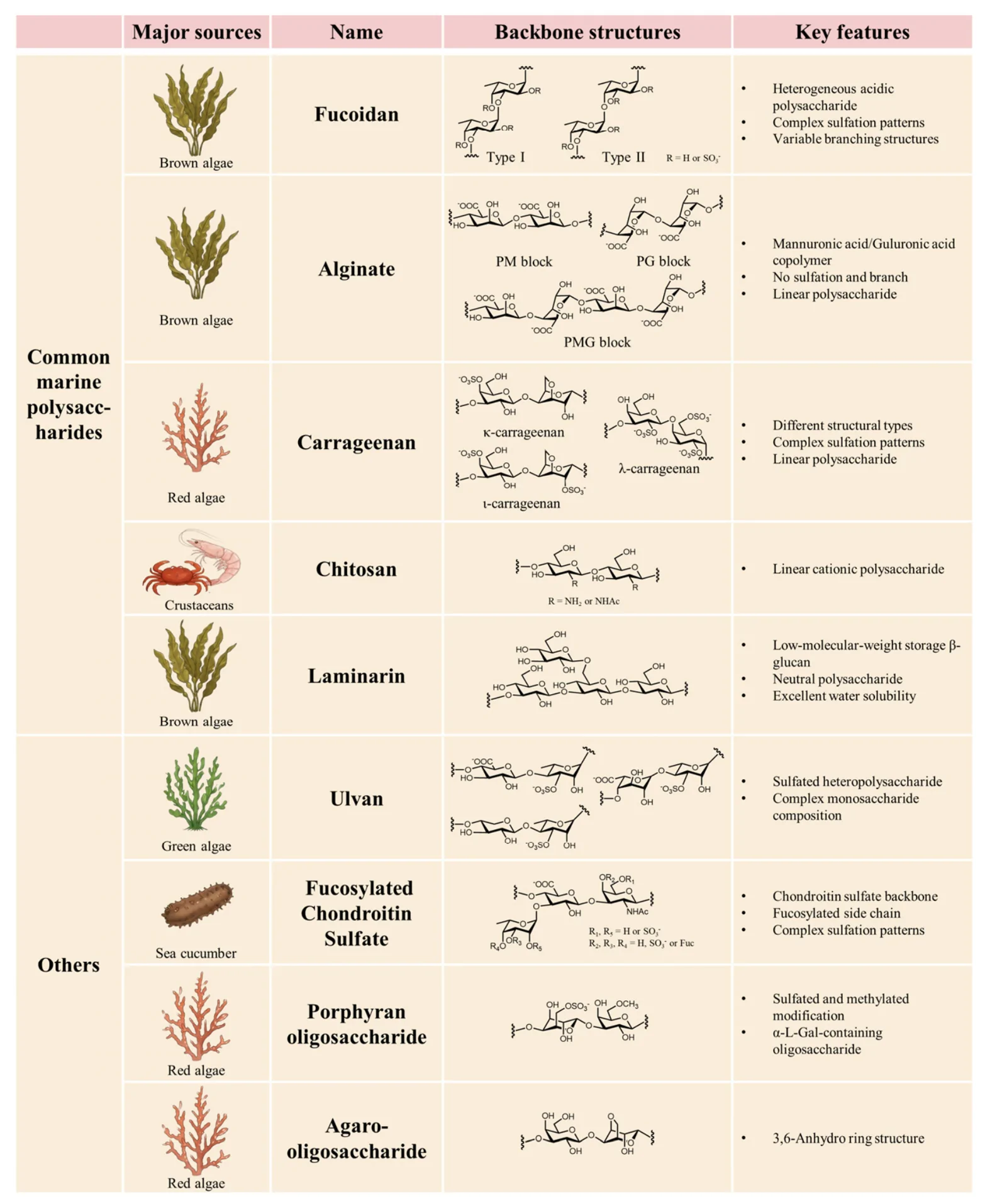
The sources, representative backbone structures, and structural features of typical marine polysaccharides.

Marine environments feature high salinity, hydrostatic pressure, low temperatures, and oxygen deficiency, which endow the distinct biological characteristics of marine organisms. Marine polysaccharides are ubiquitous and vital constituents of marine organisms, and have been widely applied in pharmaceutical, food, and cosmetic industries [22,23,24]. Common marine polysaccharides include fucoidan, alginate, carrageenan, chitosan, laminarin, and ulvan (Figure 1) [22]. Monosaccharide composition, glycosidic linkages, sulfation, and acetylation are the main features that distinguish marine polysaccharides. Their distinctive structural features endow marine polysaccharides with potential lipid metabolism-improving activity [25]. For instance, sulfation of fucoidan contributes to its strong anticoagulant activity, whereas the acetamido groups of chitosan play a vital part in its antihyperlipidemic activity.

This review summarizes recent advances in research on the antihyperlipidemic activity of marine polysaccharides and focuses on polysaccharide source and structure, structure–activity relationships, mechanisms of action, as well as application potential. We also discuss the limits and challenges in translational research, and put forward future research directions to advance marine polysaccharides into safe, effective, and sustainable natural therapeutics for hyperlipidemia.

## 2. Definition of Marine Polysaccharides

The definition and classification of marine polysaccharides are determined by their main structural features, including monosaccharide composition, glycosidic linkages, sulfation and acetylation patterns. However, the structural characteristics of each type of polysaccharide are not identical and may vary depending on the biological source, species, geographical origin, harvesting season, and physiological state of marine organisms, as well as the extraction and isolation procedures employed (Figure 1) [26,27]. Typically, extraction conditions such as thermal treatment, ultrasound-assisted extraction, microwave-assisted extraction, and enzymatic digestion can remarkably affect the structural integrity of marine polysaccharides and consequently alter their molecular-weight distribution, monosaccharide composition, and sulfate content [28,29,30]. For example, three distinct polymeric blocks (see details below) can be obtained from alginate using suitable digestion methods and isolation protocols [31]. Several strategies have been established for the depolymerization of carrageenan, including chemical and enzymatic hydrolysis, H_2_O_2_-mediated oxidative depolymerization, and ultrasonication [32]. Enzymatic hydrolysis with specific carrageenases cleaves β-1,4-glycosidic bonds in a substrate-specific manner, preventing the loss of sulfate groups and degradation of 3,6-anhydro-D-galactose moieties. In contrast, acid hydrolysis triggers random glycosidic-bond cleavage, partial desulfation, and disruption of 3,6-anhydro-ring structures [33]. In this regard, marine polysaccharides derived from different sources or prepared via distinct extraction workflows exhibit substantial structural heterogeneity, even within the same type of polysaccharide. Such structural variations can influence their physicochemical properties and biological activities and should therefore be taken into account when comparing their antihyperlipidemic effects [34]. It should be highlighted that key structural features governing the biological activity of marine polysaccharides must be primarily taken into account in bioactivity evaluation.

## 3. Fucoidan

Fucoidans are marine polysaccharides mainly derived from brown algae and several echinoderms. The backbones of fucoidans can be classified into two types: type I and type II (Figure 1) [30]. Type I backbone consist of →3)-α-L-Fuc-(1→ repeating unit, in which the partial fucose residues undergo sulfation at the 2-*O*- or 4-*O*-positions. In contrast, type II backbone are built from the disaccharide repeating motif of →3)-α-L-Fuc-(1→4)-α-L-Fuc-(1→, with sulfation occurring at the 2-*O*-, 3-*O*-, or 4-*O*-position of fucose residues. In addition to backbones and sulfate groups that are predominant structural moieties of fucoidans, the branches attached to the 2-*O*-, 3-*O*-, or 4-*O*-positions of the backbones are also critical structural characteristics of fucoidans [35]. These branched glycosyl residues generally include small proportions of galactose, xylose, mannose, glucose, rhamnose, and uronic acids.

The backbone structure, monosaccharide composition, molecular weight, branching pattern, total carbohydrate content, and sulfation pattern of fucoidans greatly depend on their biological origins and preparative techniques. For example, fucoidans isolated from marine algae typically exhibit complex monosaccharide profiles, diverse glycosidic linkages, and abundant side branches [36]. In contrast, fucoidans obtained from marine invertebrates mostly adopt linear repeating backbone structures [37]. Liu and co-workers isolated a novel fucoidan from *Sargassum fusiforme* [38]. Structural analyses showed that this fucoidan is mainly composed of →3)-α-L-Fuc-(1→, →4)-α-L-Fuc-(1→, →3,4)-α-L-Fuc-(1→, →3)-β-L-Gal-(1→, and a small amount of →6)-β-L-Gal-(1→ fragments. Sulfate groups are predominantly located at the 3-*O*- or 4-*O*-positions of Fuc residues and the 3-*O*- or 6-*O*-positions of Gal residues. This fucoidan displays remarkably distinct structural features from previously reported fucoidans. Jin, Wang and co-workers prepared a low-molecular-weight fucoidan (5.1 kDa) from *Saccharina japonica*, which features an α-1,3-linked fucose backbone with a fucose content of 47.2% and a sulfate content of 41.4% [39]. Using fucoidanase from the microbial strain *Kosakonia oryzendophytica*, Yu and co-workers enzymatically generated three low-molecular-weight fucoidan fractions designated F1 (>10 kDa), F2 (3–10 kDa), and F3 (<3 kDa) [40]. Notably, structural differences in various fucoidans significantly affect their biological activities.

Fucoidan possesses great therapeutic potential owing to its diverse biological functions, such as anticancer, antiviral, anticoagulant, antioxidant, anti-inflammatory, and antihyperlipidemic activities [41]. In particular, as a typical marine polysaccharide, the antihyperlipidemic capacity of fucoidan has attracted extensive attention [42]. Although it is well established that fucoidan exerts potent lipid-lowering effects via multiple biological pathways, including regulating lipid absorption, biosynthesis, and excretion, as well as modulating gut microbiota, the underlying mechanisms remain to be fully elucidated.

**Figure 2:**
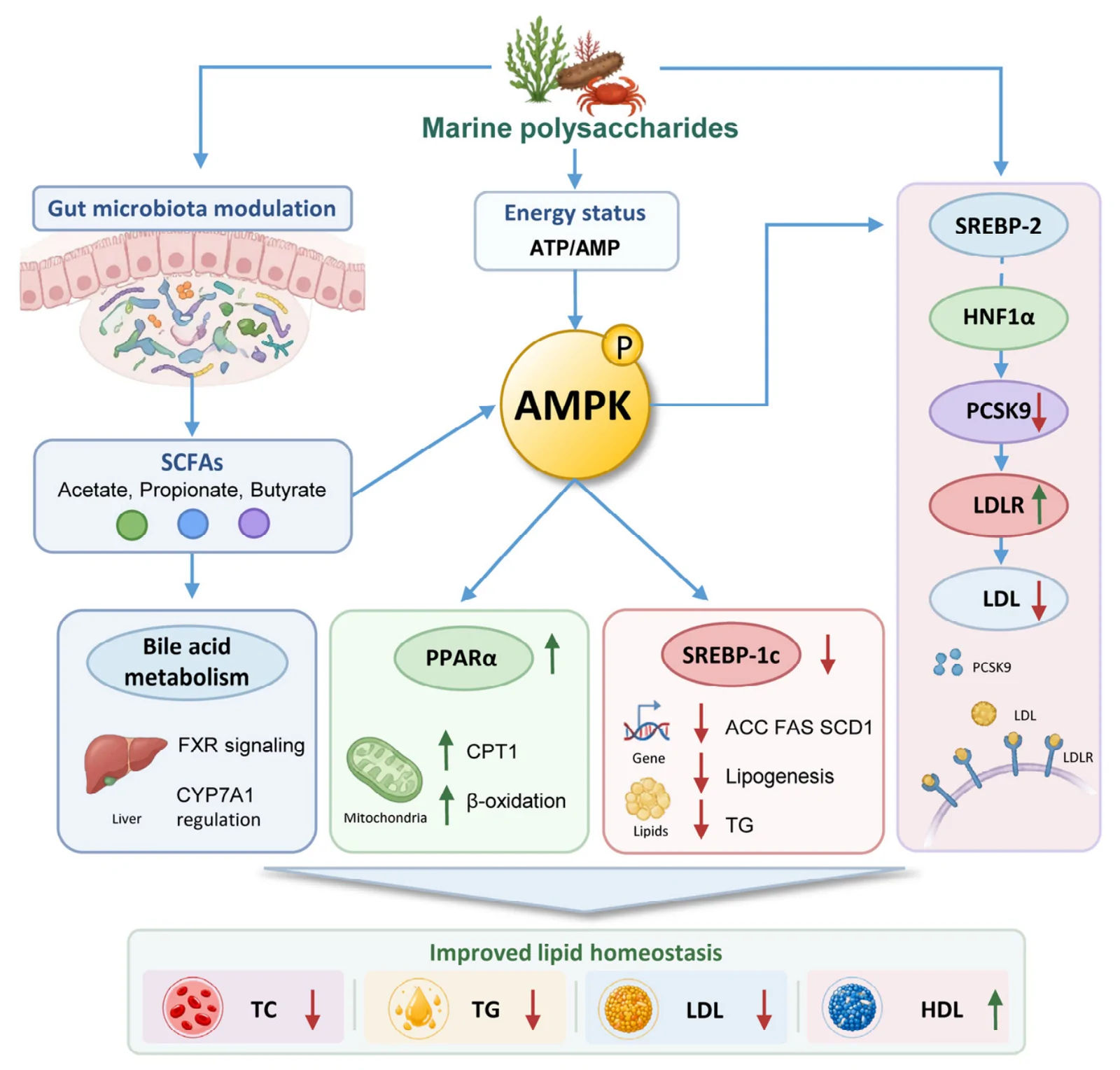
Potential mechanisms underlying the antihyperlipidemic effects of marine polysaccharides. Green upward arrows denote an increase in concentration, while red downward arrows denote a decrease.

In recent years, numerous studies have been done to decipher the lipid-regulatory mechanisms of fucoidan (Figure 2). For example, fucoidan extracted from *Saccharina sculpera* inhibits cholesterol and fatty acid biosynthesis and accelerates fatty acid β-oxidation [43]. This effect is achieved via the downregulation of HMG-CoA and sterol regulatory element-binding protein 1c (SREBP-1c), together with the upregulation of lecithin-cholesterol acyltransferase (LCAT), peroxisome proliferator-activated receptor α (PPARα), peroxisome proliferator-activated receptor γ (PPARγ), and lipoprotein lipase. Fucoidan derived from *Ascophyllum nodosum* facilitates lipid transport and metabolism through upregulating LDLR, scavenger receptor class B type 1 (SR-B1), liver X receptor β (LXRβ), cholesterol 7α-hydroxylase A1 (CYP7A1), and ATP-binding cassette sub-family A member 1 (ABCA1). In this study, oral gavage of fucoidan A3 from *Ascophyllum nodosum* (100 mg/kg/day, 4 weeks) reduced plasma TC by ~23.2% and TG by ~48.7% in high-fat diet (HFD)-fed C57BL/6J mice, surpassing the TG-lowering effect of fenofibrate (~35.5%) [44]. Furthermore, Ji, Song, and co-workers revealed that fucoidan isolated from *Fucus vesiculosus* (675.6 kDa, 23% sulfation) dose-dependently reduced intracellular cholesterol ester content in ox-LDL-induced RAW 264.7 foam cells from 55.64% (model) to 23.17% at 800 μg/mL, approaching the normal baseline of 18.61%, via transcription factor EB (TFEB)-mediated autophagic flux [45]. In addition, fucoidan acts as a prebiotic, significantly increasing the abundance of probiotic bacteria in the gut such as *Akkermansia muciniphila* and *Bacteroidetes* [46,47]. Such changes in gut microbial composition promote the production of short-chain fatty acids (SCFAs), which further suppress hepatic lipid deposition and systemic inflammatory responses. For example, fucoidan from *Sargassum fusiforme* can alleviate intestinal flora disorders to improve the lipid metabolism of high-fat diet-fed mice [38]. Fucoidan also improves intestinal barrier integrity, preventing the translocation of endotoxins that trigger metabolic inflammation [48]. In addition, a patent application (JP2001335491A) described the oral use of fucoidan or fucoidan-like sulfated polysaccharides for the prevention or treatment of hyperlipidemia, particularly hypertriglyceridemia [49]. Another patent publication (CN104523744A) described the use of fucoidan sulfate and low-molecular-weight fucoidan sulfate for lipid-lowering and weight-management applications, with reported effects on serum TG, TC, LDL-C, and HDL-C in experimental models [50]. Collectively, these findings demonstrate that fucoidan acts as a promising natural therapeutic agent for the comprehensive intervention of hyperlipidemia and its associated metabolic complications.

## 4. Alginate

Alginate is a linear anionic heteropolysaccharide predominantly extracted from brown algae and consists of β-D-mannuronic acid (M) and α-L-guluronic acid (G) in variable proportions (Figure 1) [31]. Marine-derived alginate comprises three distinct polymeric blocks: the poly-L-guluronic acid block linked via α-1,4-glycosidic bonds (PG), the poly-D-mannuronic acid block connected by β-1,4-glycosidic bonds (PM), and the alternating M/G block bound through α/β-1,4-glycosidic bonds (PMG) [31]. Furthermore, G residues of alginate adopt the ^1^C_4_ chair conformation, whereas M residues exist in the ^4^C_1_ conformation. These structural features determine the complex higher-order structure of alginate, which further accounts for differences in its biological activities. For example, PM blocks possess higher viscosity than PG blocks [51]. High-viscosity alginate can effectively alleviate hepatic lipid accumulation in rats fed a high-cholesterol diet [52]. Polyguluronate sulfate (PGS) displays markedly superior antihyperlipidemic activity compared with polymannuronate sulfate (PMS) [9].

The high molecular weight and low bioavailability greatly limit the application of alginate in food and medical industries. To address these issues, researchers have attempted to depolymerize high-molecular-weight alginate polysaccharides into alginate oligosaccharides (AOSs) [31]. Numerous studies have verified that AOS possess favorable water solubility and diverse biological activities. Three degradation methods, including acid hydrolysis, oxidative degradation, and enzymatic degradation, have been used for the depolymerization of alginate. For example, Yu and co-workers obtained low molecular weight guluronate by degrading alginate via mild acid hydrolysis (0.5 mol/L HCl, 100 °C, 8 h) [53]. Ma and co-workers degraded alginate into its low-molecular-weight product using hydrogen peroxide [54]. Alginate lyases have also been expressed and characterized for enzymatic depolymerization of alginate [55]. Notably, different degradation approaches for AOS preparation result in variations in the molecular weight, M/G ratio, and even structure of final products, which can substantially influence their bioactivities.

As the only marine-derived polysaccharide containing carboxyl groups in each monosaccharide unit, AOS and its derivatives exhibit diverse biological activities, such as antimicrobial, antitumor, anti-inflammatory, and antihyperlipidemic effects [31]. The antihyperlipidemic potential of AOS has attracted intensive attention, given that propylene glycol alginate sodium sulfate (PSS) [56], a clinically approved drug in China (the first modern marine drug approved in China), as an AOS derivative, has been widely used for nearly 40 years for the prevention and treatment of hyperlipidemia and ischemic cardio-cerebrovascular diseases. PSS is synthesized from sodium alginate via sequential hydrolysis, esterification, and sulfation. Quality control analysis showed that PSS has an M/G ratio higher than 1.5, a molecular weight of approximately 9 kDa, and a sulfation degree ranging from 9.0% to 13.0% [57]. Inspired by the successful clinical application of PSS, researchers have extensively explored the antihyperlipidemic potential and underlying molecular mechanisms of AOS and its derivatives against hyperlipidemia.

Previously, Guan and co-workers prepared propylene glycol mannuronate sulfate (PGMS) via esterification and sulfation of PM blocks [58]. PGMS is capable of reducing serum TC, TG, and LDL levels while elevating serum HDL levels in hyperlipidemic rats by upregulating the mRNA expression of LPL. Similarly, Hao, Li and co-workers synthesized propylene glycol guluronate sulfate (PGGS), a novel sulfated polysaccharide derived from alginate, which reduced intracellular TG by 36.6% and TC by 19.6% at 100 μg/mL in palmitate-induced HepG2 cells (*p* < 0.01), surpassing the TG-lowering effects of both metformin (20.0%) and lovastatin (18.4%) at their respective effective concentrations [59]. Mechanistic studies reveal that the antihyperlipidemic activity of PGGS is attributed to the activation of the AMP-activated protein kinase (AMPK) signaling pathway in PGGS-treated HepG2 cells (Figure 2). This finding is in line with an earlier study demonstrating that one pathway by which sulfated low molecular weight guluronate (SLMG) lowers TG and TC levels is to activate AMPK signaling [53]. In addition, PCSK9 and LDLR are crucial regulatory factors of circulating LDL. PCSK9 binds to LDLR on the hepatocyte surface and accelerates LDLR degradation, thereby suppressing the clearance of circulating LDL [8]. In this regard, AOS could promote the expression of LDLR through the activation of sterol regulatory element-binding protein 2 (SREBP-2) mediated by the phosphatidylinositol-3-kinase (PI3K)/protein kinase B (Akt)/glycogen synthase kinase 3β (GSK3β), and reduce the expression of PCSK9 by inhibiting hepatocyte nuclear factor-1α (HNF-1α) [60]. The formation of the PCSK9/LDLR complex is mediated by heparan sulfate proteoglycans (HSPGs) in vivo [8]. Recently, Li and co-workers found that PGS can mimic endogenous HSPGs to competitively interact with PCSK9, thus alleviating LDLR degradation on the hepatocyte surface [9]. Alginate also improves dyslipidemia by modulating gut microbial homeostasis (Figure 2) [61,62].

## 5. Carrageenan

Carrageenan is a linear sulfated polysaccharide extracted from red algae, whose structure consists of β1,4-D-galactose and α1,3-D-galactose residues (Figure 1) [63]. Based on the sulfation pattern and the 3,6-anhydrogalactose structure of repeating disaccharide units, carrageenan can be mainly divided into three types, including kappa (κ), iota (ι), and lambda (λ). The disaccharide unit structures of κ, ι, and λ-carrageenans are respectively →3)-β-(4S)Gal-(1→4)-α-anhydro-Gal-(1→, →3)-β-(4S)Gal-(1→4)-α-anhydro-(2S)Gal-(1→, and →3)-β-(2S)Gal-(1→4)-α-(2S,6S)Gal-(1→ [63]. In this regard, κ-carrageenan has the lowest sulfate ester content and the highest 3,6-anhydro-Gal content, while λ-carrageenan has the highest content of sulfate ester and the lowest 3,6-anhydro-Gal content. Generally, the molecular weight, sulfation pattern and content, and the 3,6-anhydro-Gal content collectively determine their gelation, solubility, and bioactivity. For example, the low-molecular-weight carrageenan exhibits better solubility and gastrointestinal absorption than its high-molecular-weight counterpart.

Numerous previous studies have validated the antihyperlipidemic effects of carrageenans, which can reduce serum LDL, TC, and TG, and meanwhile elevate HDL [63,64]. For example, Brown and co-workers reported that dietary supplementation with 5% *Sarconema filiforme* (a source of ι-carrageenan) for 8 weeks significantly reduced plasma TC from 1.73 to 1.51 mmol/L, restoring it to the level of healthy controls (1.59 mmol/L), and decreased body weight by ~9.5% and fat mass by ~40% in high-carbohydrate high-fat (HCHF) diet-fed Wistar rats [65]. The antihyperlipidemic mechanisms of carrageenan are multifaceted (Figure 2). Carrageenan increases the viscosity of intestinal contents to slow down intestinal digestion and nutrient absorption, thereby suppressing hepatic lipid biosynthesis and intestinal cholesterol uptake [66]. Carrageenan binds to bile salts and blocks their enterohepatic reabsorption, which further promotes hepatic bile salt synthesis and accelerates endogenous cholesterol consumption [67]. In addition, carrageenan functions as a prebiotic to modulate gut homeostasis. Long-term exposure to high-molecular-weight κ-carrageenan has been shown to reduce *Akkermansia* and *Bifidobacterium* abundance and decrease fecal butyrate levels, highlighting the need for careful evaluation of dosage and duration in clinical application [68]. Recently, Yang, Zhu and co-workers prepared the κ-carrageenan oligosaccharides from commercial κ-carrageenan by an immobilized κ-carrageenase strategy [69]. The antihyperlipidemic activity of the prepared oligosaccharides was evaluated via in vitro cell models. In vitro results revealed that these oligosaccharides significantly reduced TC, TG, and LDL levels while increasing HDL levels in HepG2 cells, and decreased intracellular free fatty acid accumulation in Caco-2 cells. Further study indicated that κ-carrageenan oligosaccharides activated AMPK signaling in hyperlipidemic HepG2 cells, upregulated the ratios of phosphorylated-AMPK/AMPK and phosphorylated acetyl-CoA carboxylase/acetyl-CoA carboxylase (p-ACC/ACC), and suppressed the expression of two key lipid-metabolism-related proteins, SREBP1 and HMG-CoA-R [69].

## 6. Chitosan

Chitosan is a linear cationic polysaccharide composed of →4)-β-N-Acetyl-D-glucosamine-(1→ and →4)-β-D-glucosamine-(1→ units, and it is the partially deacetylated product of chitin (Figure 1) [70]. The latter is primarily extracted from the exoskeletons of marine crustaceans [71]. Chitin exhibits extremely low water solubility, which limits its application in the medical industry. In contrast, chitosan bearing free amino groups is capable of dissolving in weak acids or water. The free amino cations endow chitosan with various unique physicochemical properties and biological activities [70]. For example, chitosan modulates multiple biological processes by directly interacting with negatively charged biomolecules and mucosal surfaces. High-molecular-weight chitosan can be degraded to low-molecular-weight chitosan and chitosan oligosaccharides (COSs) through acidic, enzymatic, or oxidative hydrolysis strategies [70]. Molecular weight and degree of deacetylation respectively reflect the molecular size and the quantity of cationic amino groups of chitosan, both of which determine its physicochemical properties and biological activities. For example, COSs display remarkably improved water solubility, intestinal absorbability, and systemic bioavailability [72].

Many studies have confirmed that chitosan and COSs are capable of lowering TC, LDL, and TG, and increasing the levels of HDL. Although the structure–activity relationship governing the antihyperlipidemic effects of chitosan has not been fully clarified, existing evidence supports several possible mechanistic hypotheses (Figure 2). For example, COSs alleviate hepatic lipid accumulation and lipid metabolic disorders via activating the AMPK, PPARγ, and Janus kinase 2/signal transducer and activator of transcription 3 (JAK2/STAT3) signaling pathways [73,74,75]. Recently, Je and co-workers demonstrated that COS (3.5 kDa, 90% deacetylation, 5.0 mg/mL) reduced intracellular TC, FC, CE, and TG content by 89.9%, 89.1%, 93.1%, and 76.7%, respectively, in oxLDL-induced RAW264.7 foam cells, effectively inhibiting the formation of foam cells [76], which is a major component of atherosclerotic plaques [77]. Mechanistic studies reveal that COSs suppress cholesterol influx by down-regulating the expression of class A1 scavenger receptors (SR-A1) and cluster of differentiation 36 (CD36), and promote cholesterol efflux through upregulating the expression of ABCA1 and ATP-binding cassette sub-family G member 1 (ABCG1). In addition, chitosan also exerts antihyperlipidemic effects by modulating gut microbial homeostasis [78]. For example, Shidfar and co-workers recently conducted a randomized, double-blind, controlled trial enrolling 64 overweight and obese adolescents [79]. They found that chitosan supplementation at a daily dose of 3 g for 12 weeks markedly reduced the Firmicutes/Bacteroidetes ratio from 1.48 to 0.67 (−54.7%) and enriched the populations of *Bacteroidetes* and *Akkermansia muciniphila*, confirming a close correlation between chitosan intervention and gut microbiota remodeling. In addition, a patent reported the design of alginate/chitosan-based gel systems for sequestering bile acids and long-chain fatty acids within the gastrointestinal tract, with potential applications to hyperlipidemia (JPH09241169A) [80].

## 7. Laminarin

Laminarin is a low-molecular-weight storage β-glucan predominantly consisting of β1,3-D-glucose with minor β1,6-glucose branching, which is extracted from brown algae (Figure 1) [81]. Despite the absence of carboxylate groups in its structure, laminarin displays excellent water solubility. Laminarin has an average molecular weight of approximately 5 kDa. Similar to other marine-derived polysaccharides, laminarin exerts diverse biological functions, including antitumor, immunomodulatory, and anti-inflammatory effects [82]. Its activities are influenced by the molecular weight, preparation techniques, and the degree of branching. The antihyperlipidemic potential of laminarin has not been thoroughly investigated to date.

A previous study demonstrated that laminarin supplementation affects gut microbial homeostasis in high-fat-diet-fed mice by reducing the relative abundance of *Firmicutes* while enriching *Bacteroidetes*, thereby ameliorating obesity-related metabolic disorders [83]. Sulfated laminarin can bind to heparanase, an endo-β-glucuronidase responsible for the degradation of heparan sulfate [84]. Circulating heparanase levels are elevated approximately 9-fold in patients with acute myocardial infarction compared with healthy subjects, and are associated with macrophage-rich vulnerable coronary atherosclerotic plaques [85]. Thus, laminarin treatment may suppress heparanase activity and thereby slow down atherogenesis and atherosclerotic plaque development. Recently, some efforts have been made to explore the mechanisms of action of laminarin for antihyperlipidemia (Figure 2). In 2023, Wang and co-workers demonstrated that laminarin not only markedly reduced serum TC, TG and LDL by 44.56%, 27.04%, and 40.31%, respectively, in HFD-fed C57BL/6J mice, with efficacy comparable to ezetimibe (45.99%, 33.04%, 44.72% at 10 mg/kg/day), but also increased fecal TC excretion by 37.7% [86]. Further analysis showed that laminarin is capable of down-regulating the expression of Niemann–Pick C1-like 1 protein (NPC1L1), which is a key transporter involved in dietary cholesterol uptake. Previous studies have confirmed that downregulation of senescence marker protein-30 (SMP-30) disrupts hepatic lipid homeostasis and triggers liver injury [87,88]. Recently, Park and co-workers revealed that laminarin alleviates hepatic lipid accumulation and rescues the downregulated expression of SMP-30 in the liver of high-fat-diet-induced non-alcoholic fatty liver disease mice [89].

## 8. Others

Beyond the above-mentioned marine polysaccharides, a series of less-studied marine polysaccharides have been shown to possess remarkable antihyperlipidemic potential in preclinical animal models. Ulvan, a sulfated heteropolysaccharide extracted from green algae (*Ulva pertusa)*, is mainly composed of rhamnose, uronic acid, and xylose residues (Figure 1) [90]. Ulvan can effectively decrease serum TC, TG, and LDL concentrations while increasing HDL levels in mice fed a cholesterol-rich diet [91]. Phosphorylation of ulvan can remarkably enhance its antihyperlipidemic efficacy [92]. Recently, Wang, Li, and co-workers reported that the ulvan oligosaccharide-zinc complex could activate AMPK and metal regulatory transcription factor 1/PPARα (MTF1/PPARα) signaling pathways closely associated with lipid metabolic regulation [93]. Fucosylated chondroitin sulfate (FCS) extracted from sea cucumbers consists of β-D-glucuronic acid, N-acetyl-β-D-galactosamine, and α-L-fucose units (Figure 1) [94]. FCS is well known for its potent anticoagulant activity [95]. Some studies reported the promising antihyperlipidemic activity of FCS [96,97]. For example, Chen and co-workers found that FCS is capable of improving lipid disorders by regulating lipid synthesis and lipidolysis [98]. Moreover, FCS alleviates obesity in high-fat-diet-induced mice by regulating intestinal lipid metabolism and restoring gut microbiota homeostasis [99]. In addition, porphyran oligosaccharides and odd-numbered agaro-oligosaccharides derived from red algae have also been validated to alleviate dyslipidemia in in vitro [100,101].

Collectively, dyslipidemia is a poorly understood pathological process that involves multiple systems, organs, and tissues. A typical form of dyslipidemia is hyperlipidemia, which is mainly characterized by elevated serum TC, TG, LDL, and VLDL levels, as well as decreased serum HDL concentrations [1,2]. The pathogenesis of hyperlipidemia results from the dysregulation of diverse lipid metabolic pathways, for example, excessive lipogenesis in the liver, impaired reverse cholesterol transport, gut dysbiosis, and intestinal barrier dysfunction. Moreover, several novel pathogenic signaling pathways have been identified in recent years. For example, Zheng, Jiang, Sun, Kong and co-workers revealed that long-chain ceramides bind G-protein-coupled receptors CYSLTR2 and P2RY6 to aggravate atherosclerosis [102]. Feng, Saltiel and co-workers found that dietary cholesterol can activate a Ral-dependent pathway that mediates the trafficking of cell-surface LDLR to lysosomes for degradation, a process independent of transcriptional regulation or PCSK9 [103]. Marine polysaccharides exert antihyperlipidemic effects through multi-targeted mechanisms and systemic regulation, for example, AMPK phosphorylation, upregulation of LDLR, activation of the MTF1/PPARα signaling pathway, and restoration of intestinal homeostasis (Figure 2). We firmly believe that the novel antihyperlipidemic mechanisms of marine polysaccharides remain to be explored, and these undiscovered mechanisms will surely be reported in the near future.

## 9. Limits and Challenges

**Figure 3:**
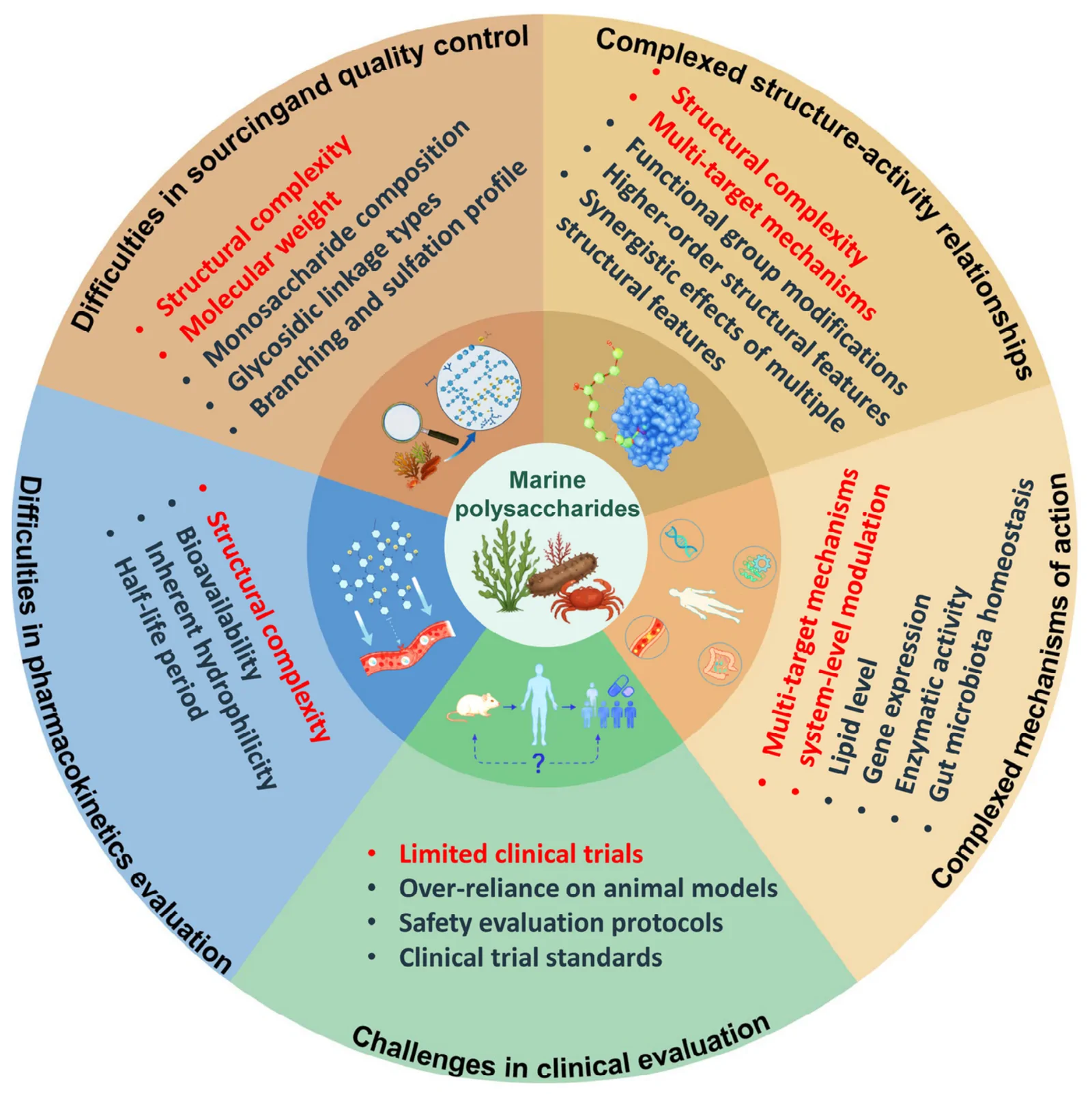
Limitations and challenges for translating marine polysaccharides into clinical antihyperlipidemic drugs.

Although marine polysaccharides exhibit promising antihyperlipidemic activity, several challenges remain to be overcome prior to their regulatory approval as antihyperlipidemic pharmaceuticals (Figure 3). Sourcing and quality control, structure–activity relationships, mechanistic elucidation, pharmacokinetic profiling, and clinical trials all require further comprehensive research to advance marine polysaccharides into reliable antihyperlipidemic therapeutics [22,104]. Notably, these challenges are common obstacles encountered in the druggability research of other natural polysaccharides [22].

Most marine polysaccharides feature prominent structural complexity and inherent microheterogeneity. Their structural variations generally manifest in molecular weight, monosaccharide composition, glycosidic linkage types, branching architectures, and sulfation profiles, which impede batch-to-batch consistency and complicate quality control. Moreover, as the primary source of marine polysaccharides, algae are affected by diverse environmental factors.

To address these challenges, the integration of conventional analytical platforms including multi-dimensional nuclear magnetic resonance and high-resolution mass spectrometry [105], advanced preparative technologies (e.g., enzymatic synthesis and cell factories) [106,107], and innovative analytical approaches such as nanopore sequencing and artificial intelligence-driven structural prediction [108,109], is indispensable for the reproducible manufacturing of marine polysaccharides. Most importantly, quality control standards should be formulated for individual marine polysaccharides.

Structural heterogeneity prevents marine polysaccharides from exhibiting well-defined structure–activity relationships. Moreover, compared with small-molecule drugs, marine polysaccharides function through multi-target mechanisms and system-level modulation, which further complicate their structure–activity relationships. Currently identified mechanisms underlying the lipid-lowering effects of marine polysaccharides involve the regulation of lipid levels, gene expression, enzymatic activity, intracellular signaling cascades, and intestinal microbiota homeostasis, the alleviation of oxidative damage, and the facilitation of cholesterol transport [110]. In this context, further studies are still needed to clarify the detailed structure–activity relationships by exploring the binding modes of polysaccharide structural moieties with corresponding biomolecular or cellular targets.

Pharmacokinetic characterization is another bottleneck restricting the druggability of marine polysaccharides. Their high molecular weight and inherent hydrophilicity result in low oral bioavailability and limited transepithelial intestinal absorption. To address these issues, novel formulations, such as nanoparticles and liposomes [111,112], have been engineered to increase the bioavailability and targeting of marine polysaccharides. For example, nanoencapsulation of fucoidan markedly improves intestinal permeability in Caco-2 cell lines and enhances its biological activity relative to free fucoidan [113]. Although marine polysaccharides generally display complex tissue distribution in vivo, fluorescence labeling and radioisotope tracing approaches have been proven capable of characterizing their biodistribution profiles [114,115]. Furthermore, available clinical evidence supporting the antihyperlipidemic potency of marine polysaccharides remains scarce. Current investigations predominantly rely on in vitro cell assays and animal models; accordingly, findings cannot be accurately extrapolated to human patients with regard to efficacy and safety profiles. Consequently, prospective clinical investigation is essential to confirm preclinical outcomes and facilitate the clinical development of marine polysaccharide-based therapies against hyperlipidemia.

## 10. Conclusions and Perspectives

Although abundant therapeutic targets have been identified and relevant drugs have entered clinical use, hyperlipidemia still drives ongoing research into innovative therapeutic alternatives. The pathogenesis of hyperlipidemia arises from dysregulated lipid biosynthesis and metabolism, processes governed by the expression of relevant genes, intracellular signaling cascades, and intestinal microbiota homeostasis. Accordingly, as natural macromolecules, marine polysaccharides possess promising therapeutic potential against hyperlipidemia owing to their distinctive physicochemical properties, multi-target mechanisms, and system-level modulation. This review outlines recent progress concerning the antihyperlipidemic efficacy of marine polysaccharides, with particular emphasis on their source and structure, structure–activity relationships, mechanisms of action, and prospective application potential. In general, marine polysaccharides have long been developed and applied as functional foods and dietary supplements to ameliorate metabolic syndrome accompanied by dyslipidemia [116,117]. Pharmacological studies indicate that, in cellular and preclinical animal models, typical marine polysaccharides including fucoidan, chitosan, laminarin, carrageenan, alginate and ulvan exhibit prominent hypolipidemic activity. Given that several marine polysaccharide-derived pharmaceuticals such as PSS and Carragelose^®^ have secured regulatory approval, while many related clinical trials, such as injectable BG136 and magnesium alginate, are currently underway, the clinical translation of marine polysaccharide-based antihyperlipidemic agents is expected in the near future [22]. In addition, with the continuous elucidation of the structure–activity relationship of marine polysaccharides in antihyperlipidemic effects, marine polysaccharide-inspired small-molecule carbohydrate drugs will achieve remarkable development.

Nevertheless, multiple bottlenecks remain unresolved in existing research, for example, sourcing and quality control, mechanistic elucidation, and pharmacokinetic profiling. Moving forward, innovative preparation workflows, analytical approaches, and tracer technologies together with advanced formulation platforms can be integrated with existing methodologies to guarantee batch-to-batch consistency, decipher structure–activity relationships and pharmacological mechanisms, and improve the pharmacokinetic characterization of marine polysaccharides.

## Data Availability

No new data were created or analyzed in this study. Data sharing is not applicable to this article.

## Abbreviations

The following abbreviations are used in this manuscript:

CVDs	Cardiovascular diseases
LDLs	Low-density lipoproteins
VLDLs	Very low-density lipoproteins
TC	Total cholesterol
TGs	Triglycerides
HDLs	High-density lipoproteins
LDL-C	Low-density lipoprotein cholesterol
PCSK9	Proprotein convertase subtilisin/kexin type 9
LDLR	Low-density lipoprotein receptors
ACC	acetyl-CoA carboxylase
HMG-CoA-R	3-hydroxy-3-methylglutaryl-coenzyme A reductase
apoB100	apolipoprotein B100
SREBP-1c	Sterol regulatory element-binding protein 1c
LCAT	Lecithin-cholesterol acyltransferase
PPARα	Peroxisome proliferator-activated receptor α
PPARγ	Peroxisome proliferator-activated receptor γ
SR-B1	Scavenger receptor class B type 1
LXRβ	Liver X receptor β
CYP7A1	Cholesterol 7α-hydroxylase A1
ABCA1	ATP-binding cassette sub-family A member 1
HFD	High-fat diet
TFEB	Transcription factor EB
SCFAs	Short-chain fatty acids
PGS	Polyguluronate sulfate
PMS	Polymannuronate sulfate
AOS	Alginate oligosaccharides
PSS	Propylene glycol alginate sodium sulfate
PGMS	Propylene glycol mannuronate sulfate
PGGS	Propylene glycol guluronate sulfate
AMPK	AMP-activated protein kinase
SLMG	Sulfated low molecular weight guluronate
SREBP-2	Sterol regulatory element-binding protein 2
PI3K	Phosphatidylinositol-3-kinase
Akt	Protein kinase B
GSK3β	Glycogen synthase kinase 3β
HNF-1α	Hepatocyte nuclear factor-1α
HCHF	High-carbohydrate high-fat
HSPGs	Heparan sulfate proteoglycans
p-ACC	Phosphorylated acetyl-CoA carboxylase
COSs	Chitosan oligosaccharides
JAK2	Janus kinase 2
STAT3	Signal transducer and activator of transcription 3
SR-A1	Class A1 scavenger receptor
CD36	Cluster of differentiation 36
ABCG1	ATP-binding cassette sub-family G member 1
NPC1L1	Niemann–Pick C1-like 1 protein
SMP-30	Senescence marker protein-30
MTF1	Metal regulatory transcription factor 1
FCS	Fucosylated chondroitin sulfate
HFFD	High-fat high-fructose diet
T2DM	Type 2 diabetes mellitus
